# Implementation Research Is Needed to Achieve International Health Goals

**DOI:** 10.1371/journal.pmed.0030186

**Published:** 2006-06-06

**Authors:** David Sanders, Andy Haines

## Abstract

Sanders and Haines discuss the "knowledge-implementation gap" and identify key obstacles to correcting the gap.

Health research needs to focus not just on the growing divide in health status between the world's rich and poor but also on the unacceptable gap between our unprecedented knowledge of diseases (including their control) and the implementation of that knowledge, especially in poor countries. Directed and innovative research is needed to analyse the causes of this situation and to point toward solutions at the global and local levels, both within and outside the health sector—given that inequitable economic globalisation is leading to greater disparities in wealth between and within countries [ 1].

Because interventions directed at health improvement require, for optimal implementation, infrastructure, equipment, supplies, and competent personnel in adequate numbers, together with intersectoral actions to address the underlying determinants of health, the term “health systems” is increasingly used. Health systems can broadly be described as containing the following principal components: structures, equipment and supplies, policies (technical priorities, financing), people (their numbers, distribution, and skills mix), and processes (how people function within the system and in relation to other sectors). How these components articulate with one another and the communities in which they are based, their effectiveness, and opportunities for modification are also framed by the social and political context in which they have evolved.

This Essay focuses on health systems research (HSR). We begin with an overview of the crisis in health, health systems, and HSR in low-income countries, with a special focus on Africa. Then, we discuss an issue that has come to be termed the “knowledge-implementation gap”, focusing particularly on those types of HSR most concerned with implementation ( Box 1). We identify some of the key obstacles to correcting this gap, and conclude with some suggestions for actions that can be taken to increase the quantity and quality of HSR.

## Weak Health Systems in Poor Countries

The gap in infant mortality and life expectancy between rich and poor countries is widening substantially. Sub-Saharan Africa is the starkest example of this growing divide. A combination of new and old infectious diseases (in particular HIV infection) and rising rates of injuries have resulted in the populations of countries such as Kenya, South Africa, Zambia, and Zimbabwe losing more than ten years in life expectancy in a short period of time [ 2]. In many of these countries, this situation is exacerbated by public health services that have been seriously weakened by chronic underfunding and loss of personnel, with an accelerating “brain drain” that is reaching crisis proportions and raising ethical questions regarding recruitment by wealthy countries [ 3, 4]. Health system dysfunction has been aggravated by ill-considered and inappropriate reforms in the health sector [ 5]. A stark reflection of these weakened health systems was the stagnation in immunisation rates over the 1990s for the six basic childhood vaccines in many poor countries, despite impressive increases in coverage during the 1980s, the availability of more and improved vaccines, and the subsequent intensive World Health Organization–driven campaigns for the eradication of polio and measles [ 6].

These challenges will require the implementation of policies that ameliorate the above underlying factors. Research can assist in achieving this but should stress health determinants, population health perspectives, HSR with a focus on implementation, and studies of the effectiveness of strategies designed to bring about equitable social and economic change.

Thus, the Mexico Statement from the Ministerial Summit on Health Research, which took place in Mexico City in November 2004 inter alia, calls on governments to allocate adequate funds to support HSR in order to address priority questions [ 7].

## Implementation Research Has Been Neglected

Health research of the types described above remains only a small fraction of global health research and a tiny proportion of expenditure on health in low-income settings. Recent estimates suggest that only about 0.017% of health expenditure in low- and middle-income countries (around US$134 million) is devoted to such research [ 8].

In public health research, the focus has traditionally been predominantly on descriptive and analytic epidemiological research (“what”, “why”, “where,” and “who”). There is growing funding for intervention research, particularly for drugs, vaccines, and other products that could benefit the poor through sources such as the Bill and Melinda Gates Foundation ( http://www.gatesfoundation.org/default.htm) and the European and Developing Country Clinical Trials Partnership ( http://www.edctp.org/default.asp?cid=68). However, there is still little funding for, and, therefore, a relative dearth of implementation research ( Box 1), particularly in low-income settings (such research addresses the “how” of translating current research knowledge into practice within health and social systems) [ 9, 10].

## Gaps between Knowledge and Action

In developed countries, implementation research focuses particularly on how to promote the uptake of research findings—for example, by evaluating a variety of strategies to enhance the use of clinical guidelines. A recent overview [ 11] suggests that different approaches might affect different behaviours. For example, reminders may be particularly appropriate for improving preventive behaviours such as immunisation and screening; feedback on performance may be effective for rationalising the ordering of diagnostic tests; and financial interventions may be effective in promoting more rational prescribing.

However, overall these effect sizes are modest, generally resulting in less than 10% improvements in practice. Combinations of a number of interventions appear to be no more effective than single interventions, perhaps because we still do not understand which combinations work best in which circumstances [ 11]. A recent review suggests that some approaches, such as supportive supervision and audit with feedback, may be effective in low-income settings, but more research is needed—not just on specific approaches to improving the quality of care, but also on the health systems environment that will sustain accessible and high-quality care over time [ 12].

HSR remains marginalised and has been dominated in the past decade by cost-effectiveness studies that have been promoted by international institutions and incorporated by governments as components of their health sector reform and rationing policies. Such research needs to be complemented by a stronger focus on the development and functioning of health systems, using a combination of quantitative and qualitative methods, including the use of action research that involves practitioners in critical reflections on their own practice. In addition, detailed and comparative case studies of the results of long-term implementation of (especially complex) interventions are needed to identify those programme and contextual factors that lead to success in health development. HSR has the powerful potential to bridge the implementation gap through testing and evaluating activities and systems while simultaneously enhancing the capacity of health staff to evaluate and improve their own performance [ 13, 14].

However, gaps between knowledge and action persist, with serious consequences for health. For example, full use of existing interventions would cut the more than 10 million annual child deaths that occur globally by more than 60% [ 15]. A high proportion of the half-million or so maternal deaths that occur globally every year could also be prevented by promoting access to interventions and services of known efficacy [ 16]. Whilst these problems are seen at their most extreme in low-income countries, they are certainly not restricted to such settings. Studies in Europe and North America show that between 30%–60% of patients do not receive effective treatment for common conditions such as asthma, heart failure, and high blood pressure. [ 17, 18].

## The Scope of Health Systems Research

Since HSR constitutes a relatively new and underdeveloped field, it is important that its scope is defined and the factors inhibiting its development are identified and addressed. A World Health Organization Task Force on HSR recently identified a number of topics for HSR ( Box 2) and made recommendations on how such research could be scaled up [ 19] (more detailed descriptions of each topic and the rationale for addressing them are given in [ 20]).

Box 3 gives an example of HSR that took place in the impoverished former Transkei “homeland” in South Africa. This example includes aspects of a number of the HSR topics listed in Box 2, such as human resources for health at the district level and below; equitable, effective, and efficient health care; and effective approaches for intersectoral engagement in health.

In some circumstances, health system interventions can be evaluated using randomised trials—particularly cluster trials, where the unit of randomisation may be communities or health facilities. A recent example is a cluster trial of a participatory intervention with women's groups to improve maternal and neonatal mortality in Nepal [ 21]. Many research questions, however, cannot be addressed by randomised trials—for example, because they may be systemwide in scope. Other approaches need to be considered, such as controlled before-and-after studies and interrupted time-series analyses and process evaluations to better understand how and why interventions work or do not work as intended. Participatory action research, which is a family of research methodologies that pursue action (or change) and research (or understanding) at the same time [ 22], has the potential to both elucidate constraints to the success of interventions and improve the performance of health staff ( Box 3) [ 23].

## Building HSR Capacity

HSR capacity is as yet limited in almost all countries. It is an interdisciplinary endeavour that demands not only technical expertise but also expertise in relating to and working with policymakers and other decision makers in developing research agendas, conducting and interpreting research, and supporting action based on the findings. While training plays an important role in developing research capacity, expertise also has to be built “on the job”, by doing research initially under supervision.

We need larger and more widely applicable research programmes that compare policies and interventions in a range of settings, assess the impact of global factors, and build HSR capacity. These could all be more easily achieved through the development of multi-country collaborative HSR networks.

At a time when substantial sums are being made available for the purchase of efficacious interventions and the development of more effective drugs, vaccines, and other products, it is essential to channel more resources to address the preparedness of health systems for delivering these interventions.

## The Next Steps

HSR is becoming recognised as a legitimate and indispensable part of health research. This has been acknowledged in, for example, the recent Mexico Summit statement [ 7]. But it is imperative to move beyond words. What, therefore, needs to happen—and who should be primarily responsible?

Educational and research institutions need to rapidly build capacity in this area of research, especially within the field of public health, since it is health systems that are the focus. These institutions need to be encouraged to do this by the creation of both financial and nonfinancial incentives. The latter come mainly from publication prestige (which, in some countries, is accompanied by financial reward to the institution or author)—hence, it is urgent that journals, especially those with high impact factors, encourage submission of articles in this area, and (where they meet the required standards) facilitate their expeditious publication. Unlike research leading to the development of pharmaceuticals, vaccines, or other health-related products, HSR has no substantial sponsorship from the private sector. Research bodies and donors can thus play an important role by calling for and funding HSR, and especially implementation research; the derisory amounts currently being spent on HSR need to rapidly increase if the benefits of much existing and new knowledge is to be realised.

Advocacy for HSR in general, and implementation research specifically, also needs to be strengthened. Policymakers can play an important role, both by demanding such research and by ensuring that health-service managers and practitioners see the value of evidence regarding the effectiveness of their activities and even acquire some skills in HSR themselves. The ongoing evaluation of the Integrated Management of Childhood Illness programme offers an indication of the potential benefits of evaluating a major international health programme that aims to promote the uptake of high-quality care based on research evidence [ 24, 25]. Such evaluations can both demonstrate the positive impacts of such programmes and highlight aspects that require further development if their full benefits are to be achieved, such as low rates of referral among children with severe illness. Presently, civil society organisations and selected research alliances are taking a lead in advocating more research in this area [ 26]. But until mainstream research organisations actively promote such research, and policymakers demand that the implementation of interventions and programmes is rigorously evaluated, the unconscionable gap between knowledge and its implementation will persist in the health field.

**Box 1. What Is Implementation Research?**

Implementation research is that subset of HSR that focuses on how to promote the uptake and successful implementation of evidence-based interventions and policies that have, over the past decade, been identified through systematic reviews. Implementation research is used as a general term for research that focuses on the question “What is happening?” in the design, implementation, administration, operation, services, and outcomes of social programmes; it also asks, “Is it what is expected or desired?” and “Why is it happening as it is?” [ 27].

In the health field, implementation research often encompasses “impact research”, which includes both research aimed at understanding what is happening during the processes of implementing changes in policy or practice, and intervention studies that are designed to compare different approaches to implementing change. Implementation research is often multidisciplinary, encompassing both quantitative and qualitative approaches that require expertise in epidemiology, statistics, anthropology, sociology, health economics, political science, policy analysis, ethics, and other disciplines.

**Box 2. Suggested Topics for HSR**

**Financial and human resources:**

- Community-based financing and national health insurance
- Human resources for health at the district level and below
- Human resources for health at the national level

**Organisation and delivery of health services:**

- Community involvement
- Equitable, effective, and efficient health care
- Approaches to the organisation of health services
- Drug and diagnostic policies

**Governance, stewardship, and knowledge management:**

- Governance and accountability
- Health information systems
- Priority setting and evidence-informed policymaking
- Effective approaches for intersectoral engagement in health

**Global influences:**

- Effects of global initiatives and policies (including trade, donors, and international agencies) on health systems

Suggested topics are from [ 20].

**Box 3. Participatory HSR Addresses Primary Health-Care Needs: Rural Hospitals, Malnutrition, and Household Food Security**

Research and development activities to improve the management of severe childhood malnutrition in rural hospitals have been continuing in the impoverished former Transkei “homeland” in South Africa since 1998. The research has involved detailed situational assessments and analyses—by paediatric ward staff, together with an outside research team—of the processes and outcomes in children admitted with a diagnosis of severe malnutrition.

The research showed unacceptably high fatality rates and serious deviations from the World Health Organization management protocol, caused by knowledge and skills deficits, inadequate resources and staff, and poor supervision and support from managers. Responses included additional resources (drugs, micronutrients, testing equipment, ingredients for special feeds, and extra night staff) and sustained training and supportive supervision, together with ongoing monitoring that is now a routine activity. This process has been successful in reducing case-fatality rates by, on average, 33% across 11 district hospitals. There is ongoing research to elucidate why some hospitals perform consistently better than others with equivalent infrastructure and resources, and indicates that differences in management and leadership are key explanatory factors.

Follow-up research of the children who were successfully treated in hospital showed that they returned to food-insecure homes, and although all households qualified for a government welfare provision to poor families (the Child Support Grant), none was receiving it, despite strenuous efforts on the part of most caregivers. Their testimony and these research findings were used in an advocacy campaign comprising formal submissions to government, newspaper articles prompting questions in parliament, and a prime-time television documentary that prompted immediate intervention by the Minister of Social Development. This, and continuing advocacy efforts in collaboration with an alliance of child-welfare nongovernmental organisations, has resulted in a sharp and sustained increase in Child Support Grant distribution and greater attention to the role of household food insecurity as a causal factor in malnutrition, although much work remains to be done.

This research illustrates the powerful potential of implementation research in developing capacity for self-evaluation—the first step in improving quality of care and in providing evidence for advocacy [ 28, 29].

## Abbreviation

HSR: health systems research
